# Dynamic changes in *Wolbachia* infection over a single generation of *Drosophila suzukii*, across a wide range of resource availability

**DOI:** 10.1002/ece3.10722

**Published:** 2023-11-15

**Authors:** Audrey E. McPherson, Paul K. Abram, Caitlin I. Curtis, Erik R. Wannop, Jan P. Dudzic, Steve J. Perlman

**Affiliations:** ^1^ Department of Biology University of Victoria Victoria British Columbia Canada; ^2^ Agriculture and Agri‐Food Canada, Agassiz Research and Development Centre Agassiz British Columbia Canada

**Keywords:** conditional mutualist, cytoplasmic incompatibility, gut microbe, insect symbiosis, larval competition, spotted wing drosophila, *Wolbachia*

## Abstract

*Wolbachia* bacteria are maternally inherited symbionts that commonly infect terrestrial arthropods. Many *Wolbachia* reach high frequencies in their hosts by manipulating their reproduction, for example by causing reproductive incompatibilities between infected male and uninfected female hosts. However, not all strains manipulate reproduction, and a key unresolved question is how these non‐manipulative *Wolbachia* persist in their hosts, often at intermediate to high frequencies. One such strain, *w*Suz, infects the invasive fruit pest *Drosophila suzukii*, spotted‐wing drosophila. Here, we tested the hypothesis that *w*Suz infection provides a competitive benefit when resources are limited. Over the course of one season, we established population cages with varying amounts of food in a semi‐field setting and seeded them with a 50:50 mixture of flies with and without *Wolbachia*. We predicted that *Wolbachia*‐infected individuals should have higher survival and faster development than their uninfected counterparts when there was little available food. We found that while food availability strongly impacted fly fitness, there was no difference in development times or survival between *Wolbachia*‐infected and uninfected flies. Interestingly, however, *Wolbachia* infection frequencies changed dramatically, with infections either increasing or decreasing by as much as 30% in a single generation, suggesting the possibility of unidentified factors shaping *Wolbachia* infection over the course of the season.

## Graphical abstract

We do not have a good understanding of how *Wolbachia* and other facultative inherited bacterial symbionts that do not manipulate host reproduction persist in their hosts; we addressed this question in a strain of *Wolbachia* that infects spotted‐wing drosophila, *D. suzukii*. To test the hypothesis that *Wolbachia* infection provides a competitive benefit when resources are limited, we established cages with varying amounts of food and seeded them with a 50:50 mixture of flies with or without *Wolbachia*. We found that although food availability did not affect *Wolbachia* prevalence, symbiont infection frequencies changed dramatically, either increasing or decreasing by as much as 30% in a single generation.

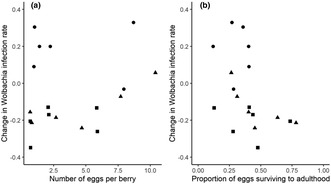

## 1 INTRODUCTION

*Wolbachia* bacteria are the most abundant host‐associated microbes on the planet, estimated to infect ~40% of terrestrial arthropod species (Zug & Hammerstein, 2012). This enormous host range is due to two distinct modes of transmission (Sanaei et al., 2021; Werren, 1997). Over ecological timescales, they are highly efficiently transmitted from females to their offspring, often in the egg cytoplasm, whereas over evolutionary timescales, they repeatedly colonize new species via mechanisms that are not well understood. In addition, many *Wolbachia* strains have evolved sophisticated strategies to manipulate host reproduction in order to increase the prevalence of infected females (Kaur et al., 2021; Werren et al., 2008). The most common of these strategies is cytoplasmic incompatibility (CI), whereby matings between infected males and uninfected females result in reduced viability of embryos. As a result, *Wolbachia*‐infected females are at a great advantage over their uninfected counterparts and can rapidly replace them (Hoffmann et al., 2011; Turelli & Hoffmann, 1991). There is currently great interest in using CI *Wolbachia* to control arthropod pests and the diseases they vector, either by using CI to rapidly drive desired traits, such as pathogen blocking, through a population (Gong et al., 2020; Hoffmann et al., 2011), or by releasing incompatible males in the wild, whereupon matings with uninfected females result in local population suppression (O'Connor et al., 2012; Zabalou et al., 2004).

Although not as well studied, many *Wolbachia* strains do not cause CI or other reproductive manipulations in their hosts. Yet, many of these strains are as prevalent and dynamic as their reproductive parasite counterparts. For example, a number of non‐CI strains of *Wolbachia* have recently invaded and spread through various *Drosophila* species (Kriesner et al., 2013; Turelli et al., 2018). In a powerful demonstration of the dynamic nature of non‐CI *Wolbachia*, Kriesner and Hoffmann (2018) used population cages to follow the prevalence of the *w*Au strain in *D*. *simulans*. Despite starting their experiment with ~35% *w*Au‐infected flies in each cage, the infection reached ~100% prevalence in only 30 generations, corresponding to an approximately 20% fitness benefit to carrying *w*Au. How and why *w*Au increases host fitness, at least under some conditions, is not known.

Indeed, condition‐dependent fitness benefits are critical in explaining the persistence of non‐CI strains, as the prevalence of maternally inherited symbionts that are not essential (i.e. obligate) for their hosts depends mainly on the fidelity of maternal transmission and the relative fitness of infected versus uninfected hosts (Hoffmann & Turelli, 1997). But the fitness benefits of non‐CI *Wolbachia* have remained largely elusive.

One potential benefit of infection with non‐CI *Wolbachia* is protection against natural enemies, with a number of *Wolbachia* strains shown to defend their hosts against pathogenic RNA viruses (Hedges et al., 2008; Teixeira et al., 2008). Interestingly, a recent study showed that wild *D*. *melanogaster* infected with *Wolbachia* were significantly less likely to harbour RNA viruses than *Wolbachia*‐free flies (Cogni et al., 2021); this is the first demonstration of strong protective effects in native host‐symbiont‐virus interactions outside of the lab.

Another possibility is that *Wolbachia* infection provides nutritional or metabolic benefits to its host, for example, by supplementing them with a limiting nutrient under stressful conditions (Brownlie et al., 2009). This was suggested as an explanation for the rapid increase in *w*Au in experimental population cages (Kriesner & Hoffmann, 2018), as flies likely experienced intense larval competition, with *w*Au‐infected larvae possibly receiving a fitness boost from their symbionts under these stressful conditions.

**FIGURE 1:**
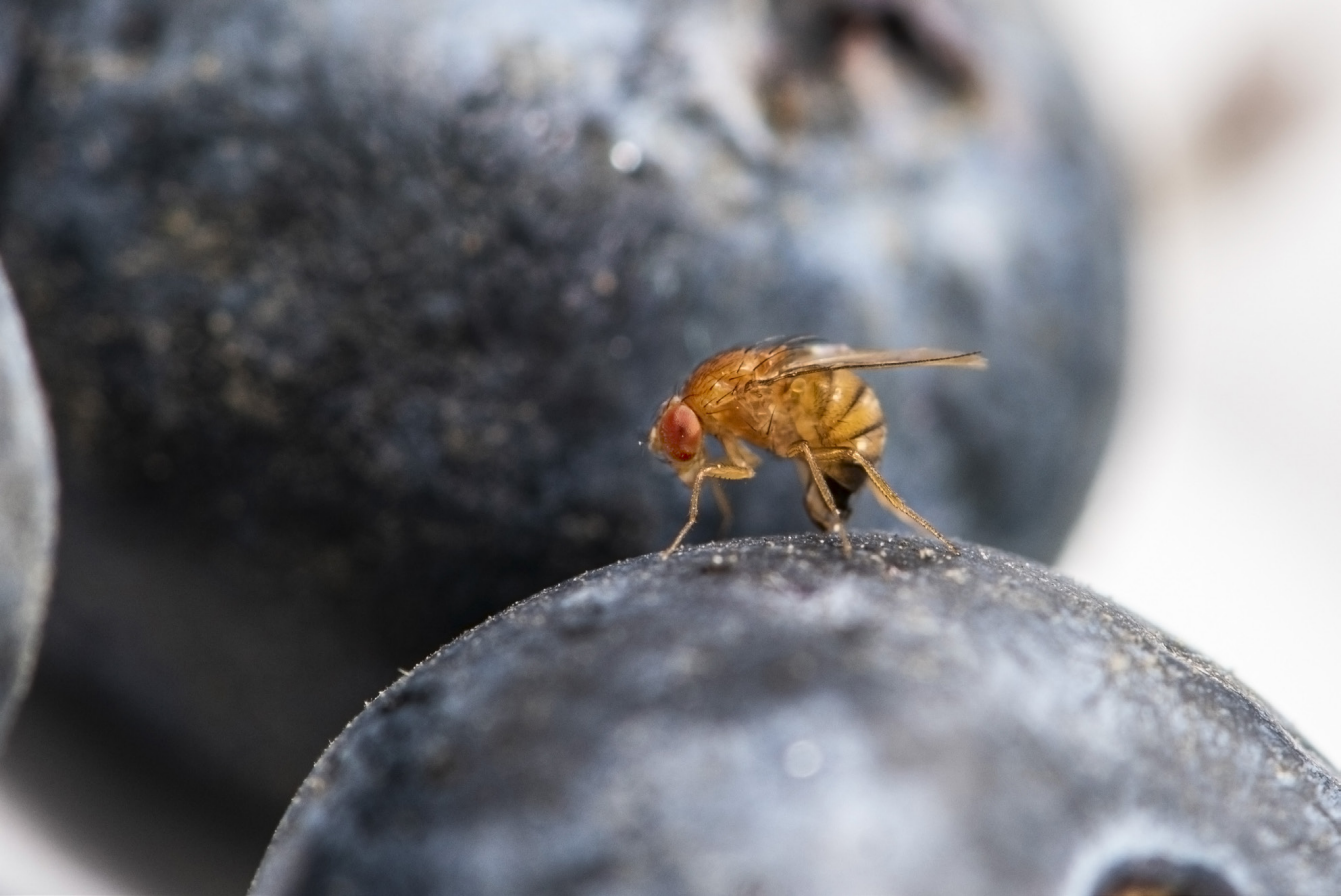
*Drosophila suzukii* ovipositing on blueberry. Photo credit: Warren Wong.

In this study, we used an experimental population cage approach to examine the dynamics of infection and conditional fitness benefits in *w*Suz, a non‐CI strain of *Wolbachia* that infects *Drosophila suzukii* (Figure 1), or spotted wing *Drosophila*, an invasive polyphagous pest of soft‐skinned fruits in both Europe and North America (Asplen et al., 2015). The *w*Suz strain does not cause CI or any other reproductive manipulations in its host (Cattel, Kaur, et al., 2016; Cattel, Martinez, et al., 2016; Hamm et al., 2014) and is imperfectly transmitted from mothers to offspring (Hamm et al., 2014). These factors alone should systematically reduce its prevalence; yet, *w*Suz infection rates are highly variable, they can be quite high in some populations but appear to average ~20% (from 7% to 57%) in North America (Hamm et al., 2014) and ~45% (from 0% to 100%) in Europe (Cattel, Kaur, et al., 2016; Cattel, Martinez, et al., 2016).

We manipulated food availability in a semi‐field setting to test the hypothesis that resource competition differentially affects the fitness of *Wolbachia*‐infected *D. suzukii*, seeding population cages with a 50:50 mixture of flies with or without *Wolbachia*. Two specific predictions arise from the hypothesis that *w*Suz boosts host metabolism under stressful and limiting conditions. First, *Wolbachia*‐infected individuals should develop more quickly than their uninfected counterparts when there is little available food. Second, there should also be greater survival of *Wolbachia*‐infected individuals, which would result in an increase in *Wolbachia* frequencies in offspring compared to their parents under higher competition scenarios.

Manipulating food availability strongly affected fly fitness. Interestingly, however, while there was no effect of resource competition on *Wolbachia* prevalence, there were pronounced and dynamic changes in infection frequency, with infections either increasing or decreasing by as much as 30% in a single generation, suggesting the possibility of unidentified factors shaping *Wolbachia* infection over the course of the season.

## 2 MATERIALS AND METHODS

### 2.1 *Drosophila suzukii* lab cultures

We established two matched *D. suzukii* lines: one *Wolbachia*‐positive, the other *Wolbachia‐*negative. The *Wolbachia*‐positive line descended from a single *D. suzukii* female collected from Chilliwack, British Columbia, Canada (49°05′52.9″ N 121°55′28.0″ W) in August of 2018 from a Himalayan blackberry, *Rubus armeniacus* (Focke) (Rosaceae). The *Wolbachia*‐free line was established from a subsample of the *Wolbachia*‐positive line treated with tetracycline (Sigma‐Aldrich) mixed with prepared Instant Drosophila Medium (Carolina Biological Supply Company) at a concentration of 0.05% for three consecutive generations. The *Wolbachia*‐positive line received identical food and environmental conditions, except that tetracycline was not added. The infection status of each line was confirmed via PCR (see below) and checked every 3–4 generations. *Wolbachia*‐positive and negative fly lines were subsequently maintained in the Perlman laboratory (University of Victoria, Canada) in an incubator (24°C; 12:12 light: dark cycle), in vials with Instant Drosophila Medium provided as a food and reproduction substrate. All flies used in the following experiments were more than 75 generations post‐antibiotic treatment. This is expected to provide more than sufficient time to re‐acquire gut microbiota and recover from the adverse effects of antibiotic treatments (Ballard & Melvin, 2007; Li et al., 2014).

For the two generations preceding semi‐field experiments, flies were reared indoors in a non‐climate‐controlled room at an average temperature of ~21°C with natural June–August photoperiods (16.2–14.8 h) in Agassiz, British Columbia, Canada. A HOBO data logger (Onset) was used to measure the temperature at 5‐minute intervals. *Wolbachia*‐positive and negative lines of flies were reared separately in 125‐mL jars (Bernardin) with 60 mL of prepared instant Drosophila medium. Three frozen blueberries were added to each jar. The tops of the jars were covered with fine insect mesh and secured with an elastic band. Each rearing jar contained 10 males and 10 females, and flies were transferred to a new jar every 3 days.

Adult flies to be used in the experiment were collected on the day they emerged and placed in a vial (diameter: 2.6 cm, height: 9.2 cm; Diamed, Canada) secured with a cellulose plug. Each of these vials contained 30 flies, consisting of 15 males and 15 females of the same age and same *Wolbachia* status. Each vial contained 15 mL of prepared Instant Drosophila Medium. One frozen blueberry was added to each vial. Flies were transferred to a new vial daily until they were 7–9 days old, when they were used in the resource competition experiment. Vials that had >50% fly mortality were not used.

### 2.2 Resource competition experiment

This experiment was designed to examine the effect of resource competition on *Wolbachia* infection frequency changes over a single generation of *D. suzukii*, and to determine whether *Wolbachia* infection increases egg‐adult development rate under a range of resource competition scenarios in semi‐field conditions.

Experiments were done during August and September of 2020, part of the seasonal period during which *D. suzukii* is actively reproducing in British Columbia (Thistlewood et al., 2019). It took place outdoors on a covered porch in Agassiz, British Columbia, Canada, exposing insects to a realistic range of abiotic conditions (photoperiod, temperature, and humidity). Temperature was recorded hourly with a HOBO logger placed among the experimental units; hourly relative humidity and daily photoperiod data were retrieved from a nearby Environment Canada weather station (Environment Canada, 2020; see Table S1 for abiotic conditions recorded during the experiment). Fresh blueberries were used as the oviposition and larval development food substrate because they are a known fruit host for *D. suzukii* (Thistlewood et al., 2019) and they are readily available throughout the summer months in British Columbia. Blueberries were stored for 48 hours at 4°C prior to use to ensure that any pre‐existing *D. suzukii* eggs or larvae were killed. The berries were then washed twice, dried, and individually inspected to ensure only undamaged berries were used.

To create different levels of resource competition, we designed three competition treatments (low, medium, and high), each of which had a consistent number of adult *D. suzukii* flies (60; 30 males and 30 females) but a varying amount of food resources (fresh blueberries) available (low – 120; medium – 60; high – 20). Of the 60 flies, 30 were from the *Wolbachia*‐infected colony and the other 30 were from the uninfected colony. Of the 30 flies from each colony, 15 were males and 15 were females. The *Wolbachia* infection status of each fly from this ‘parent’ generation was confirmed later (see below). Ventilated plastic containers lined with paper towels were used as arenas for fly oviposition. A larger container (17.1 × 25.4 × 8.1 cm; 2.12 L) was used as an oviposition arena for the ‘low’ competition treatment than the ‘medium’ and ‘high’ competition treatments (15.6 × 15.6 × 8.6 cm; 1.18 L), to ensure all blueberries were in a single layer on the bottom of each container.

The groups of 60 flies were randomly assigned to oviposition arenas with different competition treatments and allowed to oviposit for 24 hours; they were then removed and preserved in 95% EtOH. Using a dissecting microscope, the number of *D. suzukii* eggs in each berry (identified as the egg's spiracles protruding from an oviposition scar) was counted. Once the eggs were counted, all of the blueberries were placed in identically ventilated development arenas in plastic containers (15.6 × 15.6 × 8.6 cm; 1.18 L) lined with paper towels. To ensure consistent development conditions for all of the competition treatments, berries from the low and medium treatments were subdivided into multiple development arenas containing 20 berries each. Every development arena was checked daily at the same time for *D. suzukii* offspring emergence and to remove any excess condensation. As offspring emerged, they were removed from their container, their sex was noted, and they were preserved in 95% EtOH for later *Wolbachia* screening. Each development arena was kept for at least 14 days after the last observed fly emergence.

Six separate temporal blocks, each containing two replicates of each of the three competition treatments, were conducted. However, due to time constraints, DNA extraction and *Wolbachia* screening were only performed for a randomly selected three out of the six blocks, and data for only these three blocks (6 total replicates per competition treatment) are presented here.

### 2.3 Determination of *Wolbachia* infection status of parents and offspring

DNA was extracted from parental mothers and all offspring in order to determine *Wolbachia* infection status. DNA was extracted by homogenizing individual flies in 50 μL of DNA extracting buffer (9.8 mL H_2_O, 100 μL 1 M Tris pH 8.0, 20 μL 0.5 M EDTA, 50 μL NaCl) and 0.5 μL of Proteinase K (BioLabs). After the flies were homogenized, they were incubated at 37°C for 20 min, followed by an incubation at 95°C for 2 min, and stored at 4°C. Using PCR, flies were screened for *Wolbachia* using the *Wolbachia* surface protein (wsp) specific primers (wsp_81F: 5′‐ TGGTCCAATAAGTGATGAAGAAAC‐3′, wsp_691R: 5′‐AAAAATTAAACGCTACTCCA‐3′; Zhou et al., 1998) using the following thermocycling conditions: 95°C × 3 min, (94°C × 30 s, 55°C × 30 s, 72°C × 45 s) × 30, 72°C × 10 min. PCR products were visualized on a 1% agarose gel (FroggaBio) following gel electrophoresis with the use of a 1 kb plus DNA ladder (Invitrogen). *Wolbachia* status was determined based on the presence/absence of a band. A *Wolbachia‐*positive control was included along with a DNA control for *Wolbachia*‐negative samples to ensure the extraction was successful. For the DNA control, we amplified either a 708 base pair fragment of cytochrome C oxidase I (COI), a mitochondrial gene, or a 552 base pair fragment of actin, a nuclear gene. COI primers used were LCO1490 (5′‐ GGTCAACAAATCATAAAGATATTGG ‐3′) and HCO 2198 (5′‐ TAAACTTCAGGGTGACCAAAAAATCA ‐3′) (Folmer et al., 1994), and actin primers used were Act42AF (5′‐ GCGTCGGTCAATTCAATCTT ‐3′) and Act42AR (5′‐ CTTCTCCATGTCGTCCCAGT ‐3′), using the same thermocycling conditions as above, but with an annealing temperature of 58°C.

### 2.4 Data analysis

All data analysis was conducted using R version 4.2.3 (R Core Team, 2023).

We first tested whether different ratios of adult flies to food resources (number of berries) increased the number of eggs laid per berry and whether resultant resource competition resulted in decreased survival levels of immature flies. The effects of the three resource competition treatments (low, medium, and high) on the number of eggs laid in each berry (i.e., averaged across all berries in each replicate) and the proportion of eggs emerging as adults (number of adult flies emerged/number of eggs counted) in each replicate were determined by fitting generalized linear models (GLMs) with competition treatment and temporal block as categorical predictors. Poisson and binomial error distributions were used for the models with egg count and proportion survival as response variables, respectively. For the model testing the effect of competition treatment on the number of eggs per berry, a likelihood ratio test was used to evaluate statistical significance using the Anova() function in the ‘car’ package in R (Fox & Weisberg, 2019). For the model testing the effect of competition treatment on proportion emergence, to account for model overdispersion, we rescaled the statistical model by a Pearson chi‐square statistic divided by the residual degrees of freedom and used an *F*‐test to evaluate statistical significance. Temporal block was not a significant predictor of the number of eggs per berry ($\chi_{2,13}^{2}$ = 0.35, *p* = .35) or proportion emergence (*F*_2,13_ = 2.23, *p* = .15) and was not retained in the final simplified statistical models. Post‐hoc multiple comparisons among treatments were done with Tukey contrasts implemented with the glht() function in the ‘multcomp’ package in R (Hothorn et al., 2008).

Next, we tested whether changes in *Wolbachia* infection frequencies of *D. suzukii* over the single generation of our experiment (proportion of mothers infected – proportion of emerging adult offspring infected) were associated with different levels of resource competition, using GLMs with Gaussian error distributions, after verifying that model fits met assumptions of normality and homoscedasticity. Here, because there was considerable within‐treatment variation in our two metrics of resource competition intensity (number of eggs per berry, proportion of survival to adulthood; see Figure 1), we conducted these analyses with the two metrics as continuous predictor variables. ‘Temporal block’ was also included in statistical models as a categorical predictor. F‐tests were used to evaluate the statistical significance of predictor variables.

Finally, to determine whether *Wolbachia* infection status affected egg‐adult development time of *D. suzukii*, accounting for offspring sex and temporal block, a linear mixed model was fit using the package ‘lmer’ (Bates et al., 2015) with the development time of each adult fly (the number of days between replicate set‐up and the emergence of the fly) as the response variable. The initial model contained *Wolbachia* infection status (infected, uninfected), sex (male, female) and temporal block as categorical main effects, and individual container as a random effect to account for the non‐independence of the *Wolbachia*‐infected and *Wolbachia*‐uninfected individuals emerging from the same container. Model assumptions were verified by inspecting a residuals plot, and the statistical significance of each model factor was determined using *F*‐tests.

## 3 RESULTS

### 3.1 Egg density and offspring survival differed among competition treatments

**FIGURE 2:**
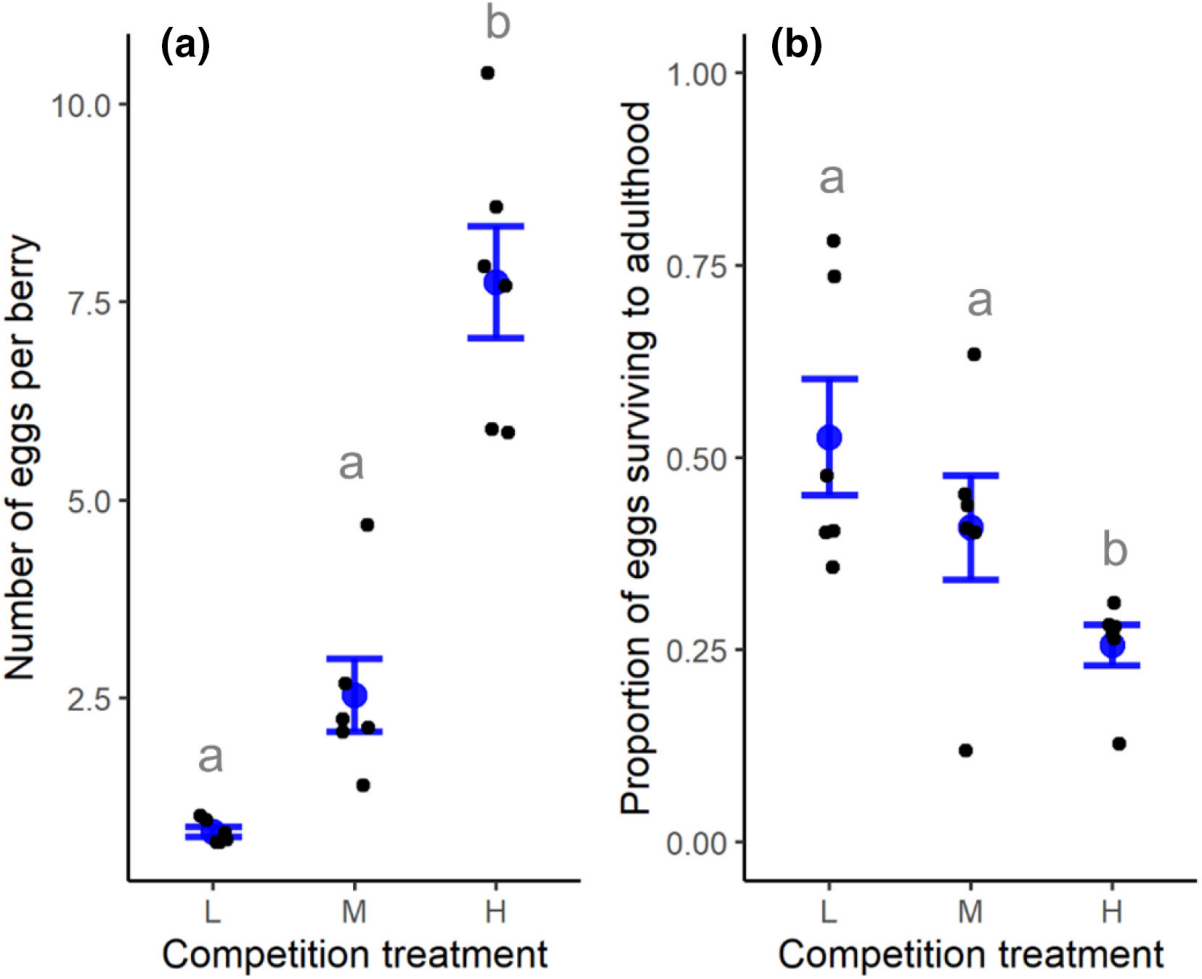
The effect of low (L), medium (M), and high (H) resource competition treatments (i.e., the relative ratio of food resources to the number of adult *Drosophila suzukii*) on: (a) the number of *D. suzukii* eggs laid per berry, and (b) the proportion of *D. suzukii* eggs surviving to adulthood. Black points represent individual replicates (*n* = 6 per treatment); blue points and error bars show treatment means with standard errors. Within panels, treatments labelled with different lower‐case grey letters are significantly different (*p* < .05; Tukey multiple comparisons on GLMs).

Increasing the ratio of adult *D. suzukii* to food resources reduced per‐offspring resource availability and resultant survival rates. Flies laid a greater average number of eggs in each berry ($\chi_{2,15}^{2}$ = 42.47, *p* < .0001; Figure 2), and a lower proportion of offspring survived (*F*_2,15_ = 6.09, *p* = .012) in the ‘high’ competition treatment compared to the ‘medium’ and ‘low’ competition treatments. Replicates with higher numbers of eggs per berry tended to subsequently have lower proportions of offspring surviving to adulthood (Pearson's correlation; *r* = −.51; *t* = −2.43, df = 16, *p* = .027).

### 3.2 *Wolbachia* infection rates can change rapidly, but are not driven by variation in competition intensity

**FIGURE 3:**
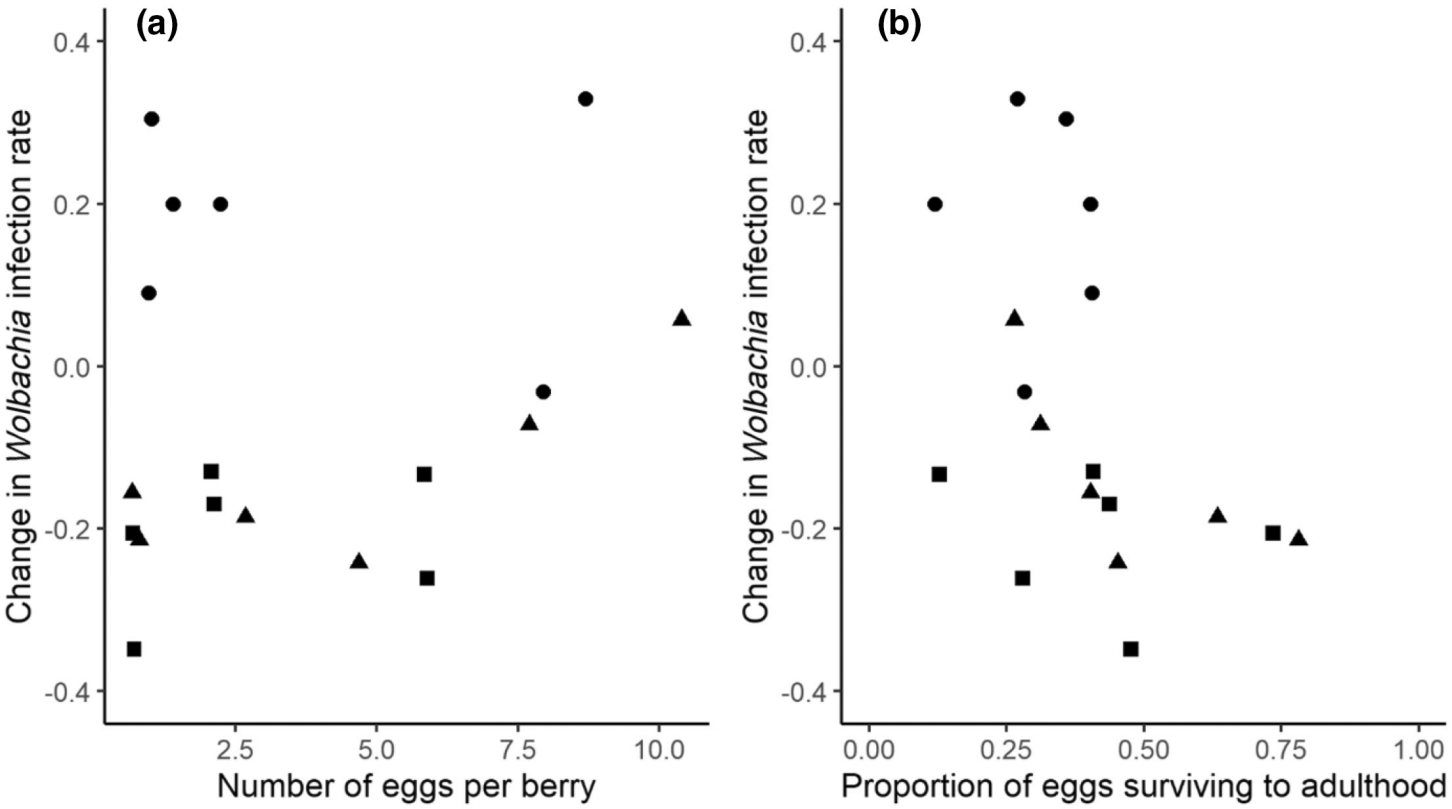
The proportional change in *Wolbachia* infection rate of *Drosophila suzukii* between parents and offspring did not vary under different levels of resource competition, measured as: (a) the initial number of eggs per berry in each replicate; and (b) the proportion of those eggs that survived to adulthood. In both panels, different symbols represent replicates belonging to different temporal blocks (circles – chronologically first block; triangles – second block; squares: third block).

Changes in *Wolbachia* infection rates between *D. suzukii* parents and offspring varied widely, from increases of 32.9% to decreases of 34.8% (Figure 3). However, the direction and magnitude of these changes among replicates were not associated with the intensity of resource competition, measured either as the initial density of eggs on fruit (*F*_1,14_ = 1.44, *p* = .25) or the proportion of eggs that survived to adulthood (*F*_1,14_ = 2.05, *p* = .17; Figure 3). The strongest and only statistically significant predictor of changes in *Wolbachia* frequency was temporal block (*F*_2,15_ = 20.52, *p* < .0001): infection rates tended to increase in the chronologically first temporal block (mean ± SE: 18.2 ± 0.06%) and decrease in the second (−13.5 ± 0.04%) and third (−20.7 ± 0.03%) blocks.

### 3.3 *Wolbachia* infection does not change offspring development time

**FIGURE 4:**
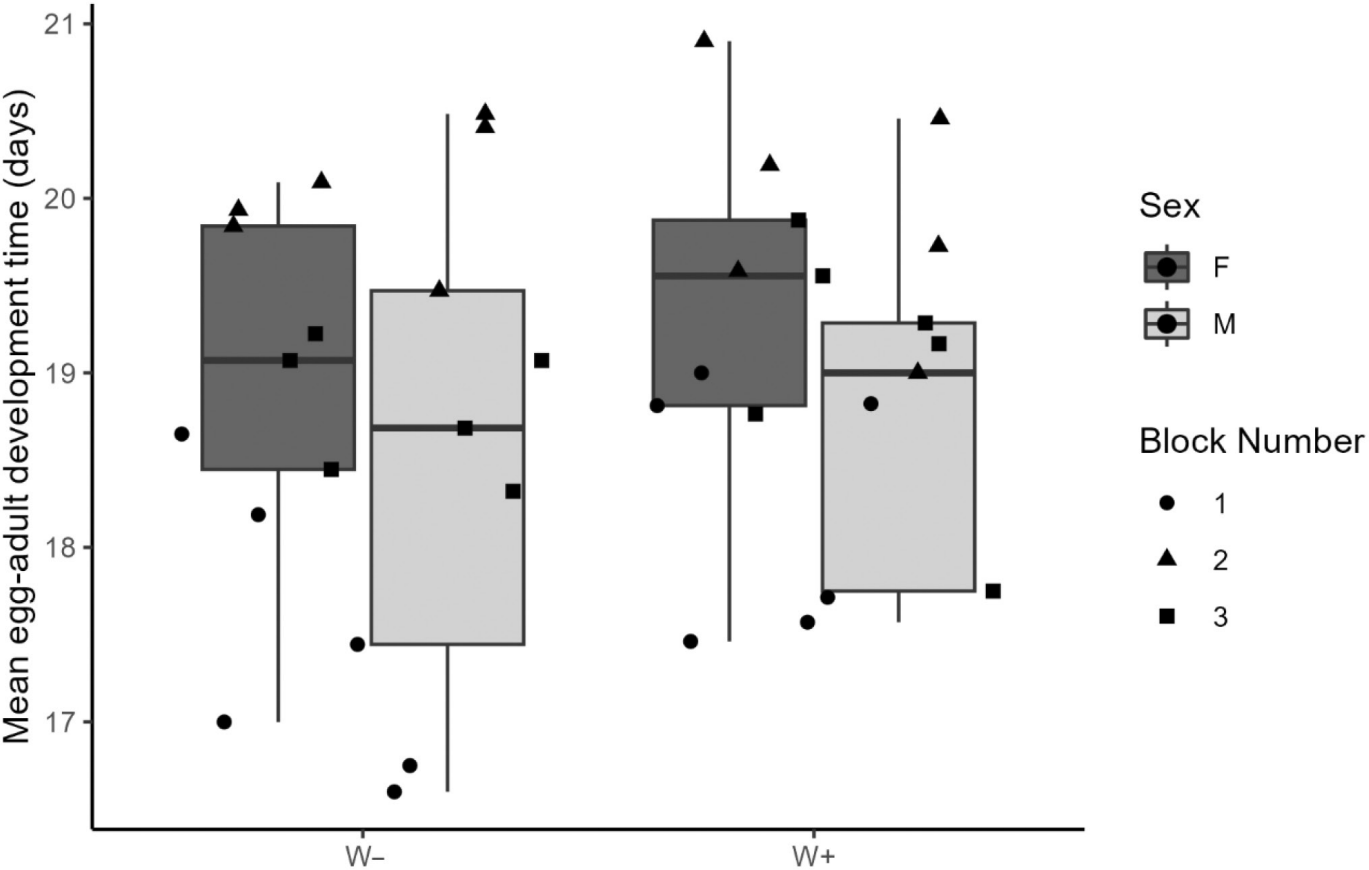
Mean *Drosophila. suzukii* offspring development times, which were marginally lower for males (light boxes) than females (dark boxes), were not associated with *Wolbachia* infection status (W+: infected; W−: not infected). Different symbols represent replicates belonging to different temporal blocks, which are numbered chronologically.

The egg to adult development time of *D. suzukii* offspring, which was marginally shorter for males than females (*F*_1,32_ = 4.27, *p* = .047; Figure 4), was not associated with their *Wolbachia* infection status (*F*_1,31_ = 2.58, *p* = .12). Development time of fly offspring varied among temporal blocks (*F*_2,32_ = 35.30, *p* < .0001), with the chronologically first temporal block having, on average, shorter development times (global mean ± SE: 17.8 ± 0.2 days) than the second (20.0 ± 0.1) and third (18.9 ± 0.2) temporal blocks.

## 4 DISCUSSION

The prevalence of maternally inherited bacterial endosymbionts is determined by a combination of how faithfully they are transmitted from an infected mother to her offspring and the relative fitness of infected versus uninfected females (Hoffmann & Turelli, 1997). A long‐standing mystery is how symbionts that neither manipulate host reproduction (or only very weakly manipulate them) nor have horizontal routes of infection, such as the strain of *Wolbachia* that infects *D. suzukii* (*w*Suz), are maintained in host populations at intermediate to high frequencies. A likely explanation is that they benefit their hosts, but only under certain conditions – but the conditions under which these benefits become apparent are often difficult to identify (Cooper et al., 2017; Harcombe & Hoffmann, 2004; Hoffmann & Turelli, 1997; Zug & Hammerstein, 2015). In this study, we tested whether *w*Suz differentially impacts host fitness under conditions of varying resource competition. While the amount of available food for developing larvae had no effect on *Wolbachia* prevalence, our major result was that symbiont frequencies were highly dynamic, changing by over 30% in a single generation. Interestingly, there were major swings in frequency in both directions, with experiments performed earlier in the summer resulting in large decreases in *Wolbachia* infection and the opposite happening in experiments performed later in the season.

Other studies have found rapid and unexplained changes in the prevalence of facultative inherited symbionts, although we are not aware of any studies that have demonstrated this in a single generation. Our study and experimental approach were inspired by Kriesner and Hoffmann's (2018) work, where the *w*Au *Wolbachia* strain increased in prevalence in population cages of *D. simulans* from 35% to over 90% in as quickly as 10 generations, corresponding to an estimated ~20% fitness benefit. The *w*Au strain is similar to *w*Suz in that it does not cause cytoplasmic incompatibility (Hoffmann et al., 1996) and is not associated with an obvious host phenotype, although a recent study suggested that it may benefit flies breeding in rotting fruits that have extensive fungal growth (Cao et al., 2019). Another noteworthy example of rapid, unexplained changes in symbiont prevalence is found in a strain of *Rickettsia* infecting *Bemisia tabaci* whiteflies in the southern United States. In just 6 years, the prevalence of infected whiteflies increased from 1% to 97% (Himler et al., 2011), and just 6 years after that, the prevalence decreased to ~35% (Bockoven et al., 2020). At the time of the increase (but not the decline), infected females had higher fitness, both in the lab and in the field, although the reason behind this fitness difference is not known.

That *Wolbachia* frequencies changed in both directions over the course of our experiment rules out the possibility that the results are due to intrinsic fitness differences – that is, that this strain of *Wolbachia*, in this host genetic background, had relatively high or low fitness effects. The flies we used in this experiment were descended from a single isofemale line, so host genetic variation was not a factor here. While we cannot completely rule out the possibility of incomplete maternal transmission of *w*Suz in our experiment, this is unlikely to have been an important factor at play here, as the infection remained stable throughout the experiment, with virtually all parents from the *Wolbachia*‐positive line infected, and *Wolbachia* frequencies increased in one of the three blocks. The *w*Suz strain that we used has been stably maintained since 2018, demonstrating that, at least in this nuclear genetic background and under lab conditions, it exhibits high maternal transmission efficiency and little fitness costs.

So what explains the dramatic changes in the *w*Suz infection? We tested the hypothesis that resource competition affects *Wolbachia*‐infected individuals differently than uninfected ones. The effect of *Wolbachia* could occur in either direction. First, if harbouring bacterial symbionts is energetically costly, we might expect that *Wolbachia*‐infected hosts would be outcompeted by uninfected ones when resources are limited. For example, *Wolbachia*‐infected *Aedes albopictus* mosquito larvae performed worse than uninfected ones when reared together at high densities but better when reared together at low densities (Gavotte et al., 2010); this may perhaps be mediated by competition between *Wolbachia* and the mosquito host over amino acids or cholesterol (Caragata et al., 2013, 2014). Another example of reduced competitive ability was found in *Trichogramma kaykai* (Huigens et al., 2004) and *T. dendrolimi* (Zhou et al., 2023) parasitic wasps, with fewer *Wolbachia*‐infected individuals emerging as adults when both infected and uninfected female wasps lay eggs in the same moth host. Alternatively, it has been proposed that *Wolbachia* may supplement its hosts with essential nutrients (Brownlie et al., 2009; Newton & Rice, 2020), such as iron, heme, riboflavin, and nucleotides, in which case we might predict that *Wolbachia*‐infected hosts would have an advantage over uninfected ones under stressful conditions. While certain strains of *Wolbachia*, such as those infecting bedbugs, have a demonstrated role as nutritional symbionts that are essential for their hosts (Hosokawa et al., 2010), we are not aware of any studies that have demonstrated competitive benefits via nutrient supplementation in facultative strains of *Wolbachia*. Regardless, while our berry density manipulations had strong effects on the number of developing flies, the relative success of *Wolbachia*‐infected individuals was not affected by competition treatment, although it is possible that imposing even more extreme resource competition may have uncovered the effects of *Wolbachia*. It would also be informative to quantify key metabolites, as well as *Wolbachia* titres, under varying conditions of resource availability.

Having ruled out a role for resource competition, we are left to speculate on what drove the large swings in *Wolbachia*. If we first consider possible abiotic factors, an obvious place to look is temperature, which has been shown to affect *Wolbachia* titres, transmission efficiency, and fitness effects in a number of *Drosophila* species (Clancy & Hoffmann, 1998; Hague et al., 2020, 2022; Saeed et al., 2018). However, there were no consistent differences in temperature between the three experimental blocks (Table S1), with the average temperature during the egg‐adult development period being almost identical (~19°C). While the temperature at the time of oviposition was higher for the second block (~25°C), it was similarly low for the first and last blocks (~16–17°C). Likewise, there were no obvious differences in humidity among blocks that correlated with changes in *Wolbachia* infection frequencies (Table S1). One notable difference between temporal blocks that did correlate with the direction of changes in *Wolbachia* infection frequencies was photoperiod, with daylength shortening over the course of the experiment (from 14.8 to 12.8 h) (Table S1). Daylength serves as an important developmental cue for *D. suzukii*, triggering major physiological changes that culminate in reproductive dormancy and increase the fly's ability to survive the winter (Hamby et al., 2016; Toxopeus et al., 2016). While we are not aware of any studies that have directly tested whether daylength affects *Wolbachia*‐host interactions, simulating reproductive dormancy resulted in lower fitness in *Wolbachia*‐infected *D*. *melanogaster* (Kriesner et al., 2016). However, work on reproductive dormancy has focused on conditions that reflect later times in the growing season than when our experiment took place, such as much cooler temperatures, and we think it unlikely that daylength drove the large changes that we observed.

Alternatively, the sometimes large swings in *Wolbachia* that we observed could have been driven by biotic factors. Infections with facultative inherited symbionts that protect their hosts against natural enemies have been shown to increase rapidly in the presence of the enemy, both in experimental population cages (Oliver et al., 2008) and in the wild (Jaenike et al., 2010). If the symbiont is costly (Vorburger & Gouskov, 2011), it may be lost if the enemy is rare or absent. Like related *Wolbachia* in *Drosophila* (Hedges et al., 2008; Teixeira et al., 2008), *w*Suz has been found to protect against pathogenic positive‐sense RNA viruses, such as the Drosophila C Virus under laboratory conditions (Cattel, Kaur, et al., 2016; Cattel, Martinez, et al., 2016; Martinez et al., 2017). A recent study of wild *Drosophila melanogaster* found that flies that harboured *Wolbachia* carried on average 0.37 fewer viruses than uninfected ones (Cogni et al., 2021). They were also approximately 3 times less likely to be infected with Motts Mill virus, a close relative of Teise virus, which appears to be widespread in *D*. *suzukii* (Medd et al., 2018), although nothing is known about how either Motts Mill or Teise viruses are transmitted or whether they are pathogenic. However, unless these viruses are highly pathogenic and commonly acquired via the food substrate (i.e. were present in the blueberries used in our study), it seems unlikely that they are responsible for the dynamic changes in *Wolbachia*.

Finally, changes in *Wolbachia* frequency may have been driven by interactions with gut microbes. Here again, we look to the work that has been done primarily in *D*. *melanogaster* to inform what may be happening in *D*. *suzukii*. A number of studies have shown that the fly's gut microbiota is dynamic, affects host fitness, and has complex interactions with *Wolbachia* (Henry et al., 2022; Henry & Ayroles, 2021; Rudman et al., 2019). For example, Henry et al. (2022) established large field enclosures of either *Wolbachia*‐positive or negative flies and sampled the fly microbiome every 2 weeks, from July to November 2019. They found rapid changes in gut microbiota composition that were also dependent on whether hosts were infected with *Wolbachia*. They also found complex interactions between *Wolbachia* and certain gut microbiota that had major effects on host fitness. For example, later in the season, *Wolbachia*‐infected flies that had abundant *Commensalibacter* bacteria in their guts were less resistant to starvation. Like *D*. *melanogaster*, the *D*. *suzukii* microbiome is dynamic, affects host fitness and is primarily acquired from food (Bing et al., 2018; Hamby & Becher, 2016; Vacchini et al., 2017), and so we suspect that the changes in *Wolbachia* frequency in our experiment may have been shaped by the microbiota. It would therefore be interesting to perform controlled experiments that manipulate the composition and abundance of blueberry and *D*. *suzukii* gut microbes, as has been done in *D. melanogaster* (Rudman et al., 2019), and to then determine how this affects the prevalence of *w*Suz.

## AUTHOR CONTRIBUTIONS

**Audrey E. McPherson:** Conceptualization (equal); formal analysis (equal); investigation (lead); visualization (equal); writing – original draft (equal); writing – review and editing (supporting). **Paul K. Abram:** Conceptualization (equal); data curation (lead); formal analysis (equal); funding acquisition (equal); project administration (equal); resources (equal); supervision (equal); visualization (equal); writing – original draft (lead); writing – review and editing (equal). **Caitlin I. Curtis:** Investigation (supporting); writing – review and editing (supporting). **Erik R. Wannop:** Investigation (supporting); writing – review and editing (supporting). **Jan P. Dudzic:** Methodology (supporting); writing – review and editing (supporting). **Steve J. Perlman:** Conceptualization (equal); funding acquisition (equal); project administration (equal); resources (lead); supervision (equal); writing – original draft (lead); writing – review and editing (lead).

## CONFLICT OF INTEREST STATEMENT

The authors declare no competing interests.

## Supporting information


Table S1

## Data Availability

Data files, R code, and a README file that describes them have been uploaded to Figshare and are currently available as private links (a single public link will be provided upon publication): Density data: https://figshare.com/s/418fc9d793c6b6b7ab10. Development time data: https://figshare.com/s/1751aec07548c7d2e9d5. R code for figures and statistics: https://figshare.com/s/d53e4b2daefa3440f09e. README file with metadata describing data and code: https://figshare.com/s/0998b81aacc25092a1ab.
