# Supplementary material for: Dynamic changes in Wolbachia infection over a single generation of Drosophila suzukii, across a wide range of resource availability
Source: Ecol Evol. 2023 Nov 15;13(11):e10722. doi: 10.1002/ece3.10722 (PMC10651314; doi:10.1002/ece3.10722)
Supplement: Supplementary file 1 — Table S1 [file ECE3-13-e10722-s001.docx]

**Table S1.** Average abiotic conditions (temperature, photoperiod, and humidity) during each of three temporal blocks.

| **Block** | **Start date - end of adult fly emergence** | **Mean ± SD** | | |
| --- | --- | --- | --- | --- |
|  |  | **Temperature (°C)** | **Relative humidity (%)** | **Photoperiod (h)** |
| 1 | August 7 - August 22 | 19.3 ± 3.0 | 73.7 ± 8.9 | 14.4 ± 0.3 |
| 2 | August 16 -August 30 | 19.2 ± 2.9 | 73.3 ± 9.1 | 13.9 ± 0.3 |
| 3 | August 26 - Sept 11 | 19.1 ± 2.3 | 66.5 ± 15.1 | 13.3 ± 0.3 |
